# Plasma uric acid level indicates tubular interstitial leisions at early stage of IgA nephropathy

**DOI:** 10.1186/1471-2369-15-11

**Published:** 2014-01-14

**Authors:** Jingjing Zhou, Yuqing Chen, Ying Liu, Sufang Shi, Xueying Li, Suxia Wang, Hong Zhang

**Affiliations:** 1 Renal Division, Department of Medicine, Peking University First Hospital, Beijing 100034, China; 2 Institute of Nephrology, Peking University, Beijing 100034, China; 3 Key Laboratory of Renal Disease, Ministry of Health of China, Beijing 100034, China; 4 Key Lab of Chronic Kidney Disease Prevention and Treatment, Ministry of Education, Beijing 100034, China; 5 Department of Statistics, Peking University First Hospital, Beijing 100034, China; 6 Laboratory of Electron Microscopy, Peking University First Hospital, Beijing 100034, China

**Keywords:** IgA nephropathy, Plasma uric acid levels, Tubulointerstitial lesions

## Abstract

**Background:**

Hyperuricemia appeared to be a common symptom in IgA nephropathy (IgAN), even in those with normal eGFR. IgAN was characterized by variation of pathological features, especially variable tubulointerstitial lesions. Since tubular reabsorption and excretion appeared to be more important in determination of plasma uric acid levels in persons without obvious decrease of glomerular filtration rate, we took advantage of our IgAN cohort to investigate whether plasma uric acid level associated with tubular interstitial lesions, and could be considered as a maker for tubular interstitial lesions, especially at early stage with normal eGFR.

**Methods:**

623 IgAN patients were involved in the present study. Morphological changes were evaluated with Oxford classification scoring system as well as Beijing classification system of IgAN. Statistical analysis was done with SPSS 13.0.

**Results:**

We found that plasma uric acid level associated with percentage of interstitial fibrosis/tubular atrophy. Higher plasma uric acid levels indicated higher tubulointerstitial scores, either with Oxford system (P = 0.012) or with Beijing classification system (P = 4.8*10^-4^) in the whole cohort. We also found that in the subgroup of 258 IgAN cases with normal baseline eGFR (eGFR > =90 ml/min/1.73 M^2^), higher plasma uric acid associated with more severe tubulointerstitial lesions with Beijing scoring system (P = 3.4*10^-5^). The risk of having more than 10% tubulointerstitial lesions in patients with hyperuricemia increased 58% compared with normal uric acid level. In subgroup with normal eGFR, only hyperuricemia predicted tubulointerstitial leisions, and the risk of having more tubulointerstitial changes increased 100%. Among these patients, hyperuricemia was associated with more tubulointerstitial lesions with a specificity of 60.3%. Specificity increased to 65% among those patients with eGFR > =90 ml/min/1.73 m^2^.

**Conclusions:**

Plasma uric acid levels indicate tubular interstitial lesions in IgAN and hyperuricemia may be considered as a marker for tubulointerstitial lesions.

## Background

Hyperuricemia appeares to be a common manifestation in IgA nephropathy (IgAN) patients, even when their glomerular filtration rate (GFR) are normal and hyperuricemia has been considered as a risk factor of chronic kidney disease progression [1-4]. However, why plasma uric acid level elevated in part of IgAN patients with young age and normal GFR did not get enough attention.

It is well known that renal handling of uric acid excretion is a major denominator of plasma uric acid levels in adults with hyperuricemia [5-7]. Renal uric acid excretion is regulated by multiple factors such as glomerular filtration rate, tubular re-absorption and excretion [5,7]. Tubular reabsorption and excretion appear to be more important in determination of plasma uric acid levels in persons without obvious decrease of GFR.

IgAN is characterized by variation of pathological features, especially variable tubulointerstitial lesions from almost normal to diffuse tubular atrophy and interstitial fibrosis [8], and tubulointerstitial damage has been reported to be an important risk factor on progression of IgAN [9-12]. So we took advantage of our IgAN cohort to investigate whether plasma uric acid level associated with tubular interstitial lesions, and furthermore could be considered as a maker for tubular/interstitial lesions, especially at early stage with normal eGFR.

## Methods

### Subjects

623 individuals (342 males and 281 females), were randomizely recruited from the IgA Nephropathy database at Renal Division of Peking University First Hospital. Among them, 258 individuals with estimated GFR (eGFR) > = 90 ml/min/1.73 m^2^. Characteristics of the population were listed in Table 1. eGFR was calculated with the equation developed from data the Chronic Kidney Disease Epidemiology Collaboration (CKD-EPI) equation [13].

**Table 1:** Baseline characteristics of the IgAN corhort

	**N = 623**	**N = 258 (eGFR > =90 ml/min/1.73 m**^**2**^**)**
Age (years)	33.2 ± 0.5	28.8 ± 0.6
Male/Female (N)	342/281	120/138
BMI (kg/m^2^)	24.8 ± 0.3	22.9 ± 0.6
SBP (mmHg)	118.7 ± 0.7	115 ± 1.1
DBP (mmHg)	74.8 ± 0.6	72.6 ± 1.0
eGFR (ml/min/1.73 m^2^)	84.2 ± 1.4	112.9 ± 1.8
pCr (μmol/L)	108.0 ± 3.0	72.8 ± 0.8
pUA (μmol/L)	374.3 ± 4.4	338.2 ± 6.7
Hyperuricemia (Y/N) (%)	51.5/48.5	37.9/62.1
TC (mmol/L)	4.9 ± 0.1	4.7 ± 0.1
TG (mmo/L)	1.5 (1.0-2.3)	1.4 (0.8-2.2)
pNa^+^(mmol/L)	140.4 ± 0.2	139.8 ± 0.2
UP (g/24 h)	1.4 (0.7-1.6)	1.3 (0.6-2.3)
Oxford score [14,15]		
M0/1 (%)	49/51	54/46
E0/1 (%)	69/31	69/31
S0/1 (%)	53/47	61/39
T0/1/2 (%)	79/10/11	95/3/2
Beijing score [16]		
T 0/1/2/3/4 (%)	7/53/19/10/11	13/68/14/3/2
Medicine (%)		
Allopurinol	0	0
Benzbromarone	0	0
Diuretics	10	0
Losartan	N/A	N/A
ACEi + ARB	42	42

Data were presented as Mean ± SEM or median (inter-quartile range [IQR]) for continuous variables and proportions for categorical variables. SBP: Systolic blood pressure; DBP: Diastolic blood pressure; UP: Urinary protein; pCr: Plasma creatinine; pNa^+^: Plasma sodium; TC: Total cholesterol; TG: Triglyceride. ACEi: Angiotensin converting enzyme inhibitor; ARB: Angiotensin receptor blocker.

The protocal for this study was approved by the Medicial Ethics Committee of Peking University and informed written consent for this study was obtained from every participant.

### Evaluation of renal pathological changes

Two pathologists re-evaluated the renal biopsy slides with Oxford classification system of IgAN. Briefly, microscopic slides of all cases were reviewed independently by each of the pathologists. Histological parameters were evaluated as: 1) Mesangial hypercellularity score (If more than half the glomeruli have more than three cells in a mesangial area, this is categorized as M1, otherwise as M0; M0 ≤ 0.5/M1 > 0.5); 2) Endocapillary hypercellularity (hypercellularity due to increased number of cells within glomerular capillary lumina causing narrowing of the lumina, E0 as absent/E1 as present); 3) Segmental glomerulosclerosis (any amount of the tuft involved in sclerosis, but not involving the whole tuft or the present of an adhesion, S0 means absent /S1 means present); 4) Interstitial fibrosis/ tubular atrophy (percentage of cortical area involved by the tubular atrophy or interstitial fibrosis, T0: 0-25%/T1: 26-50%/T2: >50%) [14,15]. Meanwhile, the histological changes of interstitial fibrosis/tubular atrophy were also graded by Beijing classification system of IgAN in present study (T0: 0%/T1: < 10%/T2: 10-24%/T3:25-49%/T4: >= 50%) [16].

### Statistical analyses

Statistical Package for the Social Sciences (SPSS v13.0; Chicago, IL) was used. Baseline characteristics were reported as mean ± SEM or median (inter-quartile range [IQR]) for continuous variables and proportions for categorical variables. Association of plasma uric acid levels and tubular atrophy/interstitial fibrosis was analyzed by univariate analysis with covariates (age, gender, BMI, proteinuria, systolic blood pressure, diastolic blood pressure and other pathological parameters) adjustment. Correlation among plasma uric acid levels, eGFR and tubulointerstitial lesions were analyzed with Spearman's correlation in the whole cohort and the subgroup with different eGFR. Logistic regression was performed to detect whether hyperuricemia was a predictor of tubulointerstitial changes. In each Model, physical and biochemical traits including age, gender, eGFR, urinary protein, systolic blood pressure, diastolic blood pressure and hyperuricemia were analyzed by single factor analysis first, and significant factors were put into multiple factor model. Chi-square was used to calculate sensitivity and specificity of hyperuricemia prediction of tubulointerstitial change.

## Results

### General data

General baseline data within one week before renal biopsy were collected. Among the 623 IgAN, 258 cases presented with normal eGFR (> = 90 ml/min/1.73 m^2^). The pathological changes of all cases were evaluated with Oxford classification system, and tubulointerstitial changes were re-evaluated with Beijing classification system as well (Table 1).

### Plasma uric acid levels associated with tubular atrophy and interstitial fibrosis

We found that plasma uric acid level associated with percentage of interstitial fibrosis/tubular atrophy. Higher plasma uric acid levels indicated higher tubulointerstitial scores, either with Oxford system (P = 0.012; Table 2) or with Beijing system (P = 4.8*10^-4^; Table 2).

**Table 2:** Plasma uric acid levels associated with tubular atrophy/interstitial fibrosis in IgA nephropathy

	**The whole cohort**		**Subgroup**	**(eGFR > =90 ml/min/1.73 m**^**2**^**)**	**P**
	**PUA (μmol/L)**	**P**	**PUA (μmol/L)**		
**Oxford score**					
T0 (N = 494)	359.2+/−4.8		T0 (N = 246)	335.9+/−6.9	
T1 (N = 59)	411.9+/−13.8	0.012	T1 (N = 8)	363.4+/−35.8	0.256
T2 (N = 70)	449.1+/−12.8		T2 (N = 4)	416.7+/−53.7	
***Tadj_Ox***					
*Tadj_Ox0*	*359.2+/−4.8*		*Tadj_Ox0 (N = 246)*	*335.9+/−6.9*	
*(N = 494)*					
*Tadj_Ox1*	*432.1+/−9.4*	1.5*10^-11^	*Tadj_Ox1 (N = 12)*	*349.8+/−2.8*	*0.150*
*(N = 129)*					
**Beijing score**					
T0 (N = 46)	340.9+/−15.5		T0 (N = 34)	335.9+/−18.3	
T1 (N = 332)	351.9+/−5.8		T1 (N = 175)	328.5+/−8.1	
T2 (N = 118)	387.0+/−9.7	4.8*10^-4^	T2 (N = 37)	371.8+/−17.6	3.4*10^-5^
T3 (N = 57)	413.7+/−13.9		T3 (N = 8)	363.8+/−37.8	
T4 (N = 70)	449.1+/−12.6		T4 (N = 4)	416.7+/−53.7	
***Tadj_Bj***					
*Tadj_Bj0*	*350.5+/−5.5*		*Tadj_Bj0 (N = 209)*	*329.7+/−7.4*	
*(N = 378)*					
*Tadj_Bj1*	*411.0+/−6.8*	*0.004*	*Tadj_Bj1 (N = 49)*	*374.2+/−15.2*	2.3*10^-4^
*(N = 245)*					

Data was presented as Mean ± SEM. Analyses were performed with univariate analysis in the whole cohort and the subgroup. PUA: plasma uric acid. Tubular atrophy/ interstitial fibrosis was analyzed as Oxford score, Beijing score separately, and adjusted by age, gender, BMI, eGFR, proteinuria, drug therapy and other pathological items. Tadj_Ox: all individuals were re-attributed into two groups, according to 25% interstitial fibrosis/tubular atrophy (Tadj_Ox0 <= 25%, Tadj_Ox1 > 25%), or to 10% interstitial fibrosis/tubular atrophy (Tadj_Bj0 < 10%, Tadj_Bj1 >= 10%).

We did the same analyses in the subgroup of 258 IgAN cases with normal baseline eGFR (eGFR > =90 ml/min/1.73 M^2^, Table 2). With Beijing classification system we also found that tubulointerstitial scores associated with plasma uric acid levels in the subgroup of normal eGFR (P = 3.4*10^-5^; Table 2), higher plasma uric acid associated with more severe tubulointerstitial lesions.

In Beijing scoring system, tubular atrophy/interstitial fibrosis more than 10% was identified as cut-off point of higher risk for ESRD [16]. Similarly more than 25% of tubular atrophy/interstitial fibrosis also increased risk of GFR decline in Oxford system. So here we re-attributed all individuals into two groups, by 10% interstitial fibrosis/tubular atrophy (Tadj_Bj0 < 10%, Tadj_Bi1 >= 10%), then by 25% interstitial fibrosis/tubular atrophy (Tadj_Ox0 < =25%, Tadj_Ox1 > 25%) and re-analyzed the association of interstitial fibrosis/tubular atrophy with plasma uric acid level, both in the whole cohort and subgroup with normal eGFR. Again, tubulointerstitial changes divided by 25% or 10% interstitial fibrosis/tubular atrophy associated with plasma uric acid levels. Patients with more tubular atrophy/interstitial fibrosis had higher plasma uric acid level (Table 2).

### Hyperuricemia as a clinical marker of tubular atrophy/interstitial fibrosis

Since higher plasma uric acid levels associated with more tubular atrophy/interstitial fibrosis, we further analyzed whether hyperuricemia was a predictor of tubular atrophy/interstitial fibrosis in IgAN. It showed that eGFR correlated better with tubulointerstitial score than plasma uric acid levels, both in the whole group and the subgroup with eGFR less than 90 ml/min. But in the subgroup with higher eGFR (> = 90 ml/min), only plasma uric acid level correlated with tubulointerstitial score (Table 3). In the logistic multivariate analysis model, variables assessed as potential predictors included age, gender, blood pressure, BMI, proteinuria, eGFR. A combined model of eGFR and hyperuricemia status predicted tubular atrophy/interstitial fibrosis in the whole cohort. The risk of having more than 10% tubulointerstitial lesions in patients with hyperuricemia increased 58% compared with normal uric acid level. In subgroup with higher eGFR (> = 90 ml/min), only hyperuricemia predicted tubulointerstitial lesions, and the risk of having more tubulointerstitial changes increased 100% (Table 4). However in the subgroup with GFR less than 90 ml/min, decrease of eGFR is the main risk factor for tubulointerstitial lesions.

**Table 3:** Correlation among tubulointerstitial score and plasma uric acid concentration in different eGFR groups

	**T Oxford score**	**Tadj_Ox**	**T Beijing score**	**Tadj_Bj**	**GFR**
***The whole cohort (N = 623)***					
**GFR**	−0.473	−0.425	−0.513	−0.451	
**p**	<0.001	<0.001	<0.001	<0.001	
**PUA**	0.262	0.257	0.284	0.261	−0.371
**p**	<0.001	<0.001	<0.001	<0.001	<0.001
***Subgroup with eGFR > =90 ml/min*/*1.73 m***^***2 ***^ ***(N = 258)***					
**GFR**	−0.082	0.031	−0.002	−0.031	
**p**	0.187	0.625	0.979	0.618	
**PUA**	0.102	0.052	0.133	0.162	−0.160
**p**	0.104	0.405	0.032	0.009	0.010
***Subgroup with eGFR < 90 ml/min*/*1.73 m***^***2 ***^ ***(N = 365)***					
**GFR**	−0.490	−0.503	−0.493	−0.378	
**p**	<0.001	<0.001	<0.001	<0.001	
**PUA**	0.234	0.240	0.243	0.199	−0.374
**p**	<0.001	<0.001	<0.001	<0.001	<0.001

Analyses were performed with Spearman's correlation in the whole cohort and the subgroup with different GFR. PUA: plasma uric acid. Tubular atrophy/ interstitial fibrosis was analyzed as Oxford score, Beijing score Tadj_Ox: all individuals were re-attributed into two groups, according to 25% interstitial fibrosis/tubular atrophy (Tadj_Ox0 < =25%, Tadj_Ox1 > 25%); Tadj_Bj: all individuals were re-attributed to 10% interstitial fibrosis/tubular atrophy (Tadj_Bj0 < 10%, Tadj_Bj1 >= 10%).

**Table 4:** Multivariate analysis for predictors of 10% tubular atrophy/interstitial fibrosis in IgA nephropathy

	**Predictors**	**OR**	**95%C.I.**	**P**
**The whole cohort (N = 623)**				
	**eGFR**	0.968	0.961-0.975	<0.001
	**HUA**	1.585	1.091-2.303	0.016
**Subgroup with eGFR > =90 ml/min/1.73 m**^**2 **^**(N = 258)**				
	**HUA**	2.097	1.112-3.957	0.022
**Subgroup with eGFR < 90 ml/min/1.73 m**^**2 **^**(N = 365)**				
	**eGFR**	0.9578	0.944-0.969	<0.001

Analyses were performed with logistic regressions in the whole cohort and the subgroup. Tubular atrophy/ interstitial fibrosis were analyzed as two groups, less than 10% interstitial fibrosis/tubular atrophy or more than 10% lesion (Tadj_Bj0 < 10%, Tadj_Bj1 >= 10%). In each Model, physical and biochemical traits including age, gender, BMI, baseline-eGFR, baseline UP, SBP, DBP, drug therapy and hyperuricemia status were analyzed by single factor analysis first, and factors that were significant were then put into multiple factor model. Result by multiple factor analysis was presented. HUA: hyperuricemia (male >420 μmol/L, female >360 μmol/L); eGFR: estimated GFR.

Among these patients, hyperuricemia associated with more tubulointerstitial lesions with a specificity of 60.3%. Specificity increased to 65% among those patients with eGFR > =90 ml/min/1.73 m^2^ (Table 5).

**Table 5:** Specificity and sensitivity for predicting tubular atrophy/interstitial fibrosis by hyperuricemia in IgA Nephropathy

	**Sensitivity**	**Specificity**	**P (Chi-square)**	**P (Exact)**
**The whole cohort**				
**HUA**	62.0%	60.3%	4.9*10^-8^	5.5*10^-8^
**The subgroup with normal eGFR (> = 90 ml/min/1.73 m**^**2**^**)**				
**HUA**	53%	65.1%	0.016	0.022

HUA: hyperuricemia.

## Discussion

Tubulointerstitial damage has been reported to be an important risk factor for progression of IgAN [9-12]. Many IgAN patients have tubular atrophy and interstitial fibrosis even with normal eGFR. And it appeared that pathological examination was the only method to detect renal tubulointerstitial lesions. Hyperuricemia has been an interesting clinical symptom in IgAN patients and considered to be associated with prognosis of the disease [1,17,18]. As early as 1975, Berger et al. has reported that 20-60% patients with gout also have mild to moderate renal dysfunction [19]. Besides influence of glomerular filtration rate on elimination of uric acid, several studies suggested that renal tubules play crucial roles in the regulation of uric acid balance in the body [20,21]. Thus we systematically evaluated the pathological changes of 623 individuals with IgAN by Oxford classification system [14,15], as well as the classification for tubulointerstitial changes in Beijing system [16] and found that tubular atrophy/interstitial fibrosis associated with plasma uric acid levels. The higher plasma uric acid levels indicated higher tubulointerstitial scores. And we also found similar association in the subgroup with normal eGFR. Finally, we identified that hyperuricemia could be a marker to predict tubular atrophy/interstitial fibrosis in IgAN, especially in those with normal eGFR.

In our study, we took advantage of the new pathological classification system, Oxford system [14,15] as well as Beijing system [16] and did analysis in whole cohort of 623 IgAN patients, then in subgroup with eGFR > =90 ml/min/1.73 m^2^ and subgroup with eGFR < 90 ml/min. Our result showed that eGFR correlated with tubulointerstitial score better than plasma uric acid levels in the whole cohort and in the subgroup with eGFR less than 90 ml/min. Lower eGFR correlated with severe tubulointerstitial lesions. However in the subgroup with eGFR > = 90 ml/min, eGFR did not correlate with tubulointerstitial score anymore, only uric acid levels presented correlation with the scores. Previously published reports also found that plasma uric acid correlated with pathological changes in 202 cases of IgAN, in which the best correlation coefficients were 0.42 for interstitial fibrosis and 0.39 for tubular atrophy [17]. Although our study did not identified such high correlation coefficients for plasma uric acid and tubulointerstitial lesions, we found that plasma uric acid but not eGFR was an factor associated with tubulointerstitial changes in patients with obviously normal eGFR. However when eGFR decreased, eGFR itself may be correlating with tubulointerstitial changes.

Since tubular atrophy/interstitial fibrosis more than 10% was identified as cut-off point of higher risk for ESRD in Beijing scoring system [16] and more than 25% tubular atrophy/interstitial fibrosis increased risk of GFR decline in Oxford system, we collapsed individuals into two groups by cut-off point at 10% for Beijing scoring system, then by 25% interstitial fibrosis/tubular atrophy for Oxford scoring system. We set up two models of multivariate logistic regression, in which dependent variable was tubulointerstitial lesion by 10% cut off in model 1 and tubulointerstitial lesion by 25% cut off in model 2. For model 1, the risk of having more than 10% tubulointerstitial lesions in IgAN patients with hyperuricemia increased compared with normouricemia in whole cohort as well as in subgroup with normal eGFR. For model 2, none of the clinical signs predicted tubulointerstitial changes. These may be explained by the time of renal biopsy. Most patients accepted renal biopsy were attributed to less than 25% tubulointerstital lesions group, especially those with normal eGFR.

Our study showed that hyperuricemia has specificity of 60-64% to be associated with tubulointerstitial lesions in IgAN. Although it is much less than accepted levels of specificity 80% to consider hyperuricemia a predictor for tubulointerstitial lesions, the specificity still indicated the close association of hyperuricemia with tubulointerstitial score.

In summary, this observation study at an IgAN cohort revealed that interstitial fibrosis/tubular atrophy was associated with plasma uric acid levels in IgAN and plasma uric acid level was a hopeful clinical marker indicating tubulointerstitial lesions especially in patients with normal eGFR.

## Conclusions

Hyepruricemia appeared to be a common symptom in IgA nephropathy. We took advantage of our IgAN cohort and found that plasma uric acid level indicates tubulointerstitial lesions in IgAN and hyperuricemia may be considered as a marker for tubulointerstitial lesions.

## Competing interest

The authors declare that they have no competing interest.

## Authors’ contributions

CYQ conceived of the study, and participated in its design and coordination and helped to draft the manuscript. ZJJ collected the clinical data and drafted the manuscript. LY participated in the collection of clinical data. SUF and WSX re-evaluated the renal biopsy slides with Oxford classification system and Beijing classification system of IgA nephropathy. LXY helped to perform the statistical analysis. ZH participated in the design of the study. All authors read and approved the final manuscript.

## Pre-publication history

The pre-publication history for this paper can be accessed here:

http://www.biomedcentral.com/1471-2369/15/11/prepub
